# Type 2 Diabetes : An Independent Risk Factor for Tuberculosis: A Nationwide Population-Based Study

**DOI:** 10.1371/journal.pone.0078924

**Published:** 2013-11-13

**Authors:** Ming-Chun Kuo, Sheng-Hao Lin, Ching-Hsiung Lin, I-Chieh Mao, Shun-Jen Chang, Ming-Chia Hsieh

**Affiliations:** 1 Department of Internal Medicine, Kaohsiung Chang Gung Memorial Hospital, Kaohsiung, Taiwan; 2 Chang Gung University College of Medicine, Kaohsiung, Taiwan; 3 Division of Chest medicine, Department of internal medicine, Changhua Christian Hospital, Changhua City, Taiwan; 4 Department of respiratory care, College of health sciences, Chang Jung Christian University, Tainan, Taiwan; 5 Taiwan School of Medicine, Chung Shan Medical University, Taichung, Taiwan; 6 Graduate Institute of Biomedical Sciences, National Chung Hsing University, Taichung, Taiwan; 7 Division of Endocrinology and Metabolism, Department of Internal Medicine, Changhua Christian Hospital, Changhua, Taiwan; 8 Department of Kinesiology, Health and Leisure Studies, National University of Kaohsiung, Kaohsiung City, Taiwan; 9 Graduate Institute of Integrated Medicine, China Medical University, Taichung, Taiwan; Fundacion Huesped, Argentina

## Abstract

**Objective:**

Tuberculosis continues to be a major global health problem. We wanted to investigate whether Type 2 diabetes was a risk factor for tuberculosis in an Asian population.

**Methods:**

From Taiwan’s National Health Insurance Research Database, we collected data from 31,237 female patients with type 2 diabetes and 92,642 female controls and 32,493 male patients with type 2 diabetes and 96,977 male controls. Cox proportional hazard regression was performed to evaluate independent risk factors for tuberculosis in all patients and to identify risk factors in patients with type 2 diabetes.

**Results:**

During the study period, both female (standardized incidence ratio (SIR): 1.40, p<0.01) and male (SIR: 1.48, p<0.01) patients with type 2 diabetes were found to have a significantly higher rate of incident tuberculosis than the control group. Type 2 diabetes (HR:1.31, 1.23–1.39, p<0.001) was significantly associated with tuberculosis after adjusting sex, age, bronchiectasis, asthma and chronic obstructive lung disease.

**Conclusions:**

Patients with type 2 diabetes have a higher risk of tuberculosis compared to control subjects after adjusting for confounding factors. The current diabetes epidemic may lead to a resurgence of tuberculosis in endemic regions. Therefore, preventive measures, including addressing the possibility that type 2 diabetes increase the individual’s susceptibility for incident TB, should be taken to further reduce the incidence of tuberculosis.

## Introduction

With a high prevalence and approximately 9.4 million new cases and 1.7 million deaths each year, tuberculosis (TB) remains a high global health threat and a World Health Organization (WHO) concern [1]. Although the incidence rate of tuberculosis has been decreasing in many parts of the world, current decreases are not consistent with WHO’s Global Plan to stop TB target by 6% per year, and their target of eliminating tuberculosis by 2050 will not be achieved [2]. Currently proposed strategies to improve the control of tuberculosis include targeting specific high-risk groups, meaning that it is essential to identify and characterize the determinants of tuberculosis risk so that resources and interventions can be directed at those most likely to develop and transmit tuberculosis.

Type 2 diabetes mellitus (DM) is a very common medical disorder and the leading cause of morbidity and mortality worldwide. There are 285 million cases of DM per year, and that figure is expected to reach 438 million cases by 2030 [3]. The greatest increases in prevalence of diabetes are occurring in developing countries [3] where tuberculosis is endemic. Therefore, a better understanding of relationship between diabetes and tuberculosis might be helpful in determining the most effective public health measures to curb the potential convergence of these epidemics.

Although previous studies have found an association between diabetes and tuberculosis, most of these were case-control studies [4]–[7], which may not be a valid reflection of the true risk of tuberculosis in association with diabetes. We used Taiwan’s National Health Insurance Research Database (NHIRD) to conduct a longitudinal cohort study to investigate whether type 2 diabetes is a risk factor for incident tuberculosis in this Asian population.

## Materials and Methods

### Source Population and Data

The National Health Insurance (NHI) medical claims database, including ambulatory care, hospital inpatient care, dental services, and prescription drugs, is managed by Taiwan’s National Health Research Institutes (NHRI). The NHRI provided a database of 1,000,000 random subjects for this study, representing about five percent of Taiwan’s population. There are no significant differences in age, sex, and characteristics between this same and the entire population of enrollees. For this study, we obtained a longitudinal cohort from the NHIRD 2000 to 2011. The approval for analysis of the database was obtained from the Institutional Review Board of Changhua Christian Hospital (CCH IRB 121213).

### Study Sample

NHI diagnosis coding follows the International Classification of Diseases, Ninth Revision (ICD-9), Clinical Modification diagnostic criteria. We included patients with type 2 diabetes diagnosed with the ICD-9 (coding 250 and excluding type 1 diabetes with ICD-9 code 2501) after 1 January 2000. The diagnosis was then further confirmed by continuous prescriptions of antidiabetic medications for > = 60 days. The control group was composed of patients without Type 2 diabetes or tuberculosis. We randomly selected three non-diabetic controls matched to each diabetic patient by gender, year of birth, and month and year of first diagnosis at enrollment. This matching ensured that sex, age, and follow-up time were nearly the same between study subjects and controls. We excluded those who had diagnosis of Systemic Lupus Erythematosus (SLE), human immunodeficiency virus (HIV) infection, and lung cancer in patients with type 2 diabetes and control subjects.

### The Endpoint and Follow Up

The primary endpoint was the occurrence of tuberculosis identified as the presence of ICD-9 (010–018). The diagnosis was then further confirmed by a continued dispensing of anti-TB drugs for > = 60 days. The occurrence of tuberculosis must be at least 12 months later after the diagnosis of type 2 diabetes. Diseases diagnosed before the onset of tuberculosis were analyzed as possible confounders. They were TB attacker, bronchiectasis, chronic obstructive lung disease (COPD), and asthma as well as age and sex. The following diseases were indicated by ICD-9 codes: TB attacker (799.5), bronchiectasis (494), SLE (710.0), HIV (042), COPD (491, 492, 494 or 496) and asthma (493). TB attackers were defined as subjects who had contact with patients with tuberculosis at the frequency of more than 40 hours a week.

### The Incidence of Tuberculosis and Standardized Incidence Ratio (SIR)

We estimated the incidence of TB per 1000 person-years in patients with type 2 diabetes and control subjects, and calculated the standardized incidence ratio (SIR), which is risk ratio of incident tuberculosis in patients with type 2 diabetes as compared to the controls [8].

We used the following definitions and formulae for attributable fractions (AF) [9].

### Attributable Fraction (Population)

The proportion by which the incidence rate of the outcome of interest (here, incident tuberculosis) in the entire population would theoretically be reduced if the exposure of interest (here, diabetes) were eliminated.
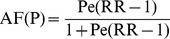
Where P_e_ is the prevalence of the exposure and RR is the relative risk for the outcome of interest.

### Attributable Fraction (Exposed)

The proportion by which the incidence rate of the outcome of interest (here, incident tuberculosis) in the exposed population would theoretically be reduced if the exposure of interest (here, diabetes) were eliminated.
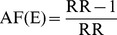
where RR is the relative risk for the outcome of interest.

We calculated crude estimates of the AF(P) using data for tuberculosis incidence and diabetes prevalence for the overall population stratified by sex.

### Measurement of Covariates

We considered the following variables previously identified as risk factors for incidental tuberculosis as covariates: sex, age, COPD, asthma, and brochiectasis.

### Statistical Analyses

Cox regression models were fitted to estimate the effects of type 2 diabetes controlling for gender, age, COPD, asthma, and brochiectasis. The hazard ratio (HR) and 95% confidence intervals (95% CI) used to test significance. The cumulative HR for incident in patients with type 2 diabetes compared to the controls was estimated by Log-Rank test. All statistical operations were performed using SAS program (v9.3) after mining the national outpatient records using the PERL (v5.8) program.

## Results

This study included a total of 31,237 female patients with type 2 diabetes and 92,642 female controls and 32,493 male patients with type 2 diabetes and 96,977 male controls, all found from the outpatient database for the period of 2000–2011. The mean ages of case and control groups were 56.79 (±12.39) and 56.67 (±12.26) years for females and 54.30 (±12.62) and 54.23 (±12.58) years for males, respectively (Table 1). Table 1 analyzes the distribution by age and diabetes status among the females and males. The frequency distribution of type 2 diabetes and the controls was not significantly different in each age segmentation in either males or females.

**Table 1:** The frequency distributions among gender in patients with type 2 diabetes and control groups stratified by age.

Gender	Females		Males	
	Type 2 diabetes	The controls	Type 2 diabetes	The controls
Number	(n = 31237)	(n = 92642)	(n = 32493)	(n = 96977)
Age (means ± SD; years)	56.79±12.39	56.67±12.26	54.30±12.62	54.23±12.58
**Age Groups**	**n (%)**	**n (%)**	**n (%)**	**n (%)**
> = 18, <30	748 (2.39)	2237 (2.41)	792 (2.44)	2393 (2.47)
> = 30, <40	1988 (6.36)	6019 (6.50)	3240 (9.97)	9699 (10.00)
> = 40, <50	6043 (19.35)	18072(19.51)	8567 (26.37)	25655 (26.45)
> = 50, <60	9557 (30.60)	28390 (30.64)	9396 (28.92)	28141 (29.02)
> = 60, <70	8530 (27.31)	25339 (27.35)	6580 (20.25)	19629 (20.24)
> = 70	4371 (13.99)	12585 (13.58)	3918 (12.06)	11460 (11.82)
Total	31237 (100)	92642 (100)	32493 (100)	96977 (100)

A total of 5103 cases of incidental tuberculosis were diagnosed during the follow-up period. Three hundred and nine patients were diagnosed with extrapulmonary tuberculosis (6.06%), and the remaining were diagnosed with pulmonary tuberculosis (93.94%). The cumulative incidence of TB was estimated to be 1.92 cases per 1000 person-years among female diabetes cases during the 2000–2011 period; female controls had an incidence of 1.37 cases (Table 2). Age-adjusted SIR was 1.40 (95% CI = 1.24–1.58), which is to say that women with diabetes had 1.40 times of the risk of developing TB compared to controls after adjusting for age. Furthermore, the rate ratio of incidences of TB among these case-control groups calculated for different age groups showed that female patients with type 2 diabetes aged 30–39 had high RR values (RR = 1.83), which decreased gradually by decade 1.25 in those aged 60–69. There was no significant increase in RR of incident TB in diabetic females aged more than 70 years old compared to the controls. The incidence of TB among males with diabetes per 1000 person-years was 3.25 cases, compared to 2.19 cases in male controls. SIR was 1.48 (95% CI = 1.35–1.63) after adjusting for age (Table 3). The rate ratios (RR) of incidences of TB showed significant association type 2 diabetes and TB in men aged between 30 and 70 years (RR between 2.21-1.23), though no significant result was found in men aged 18–30 years or men 70 years old or older. The attributable fraction (population)% among females and males were estimated to be 3.03 and 4.09, respectively. The attributable fraction (exposed)% among females and males were estimated to be 28.6 and 32.6, respectively.

**Table 2:** The age-adjusted standardized incidence ratio of incident tuberculosis among female diabetic patients stratified by age.

	Type 2 diabetes (n = 23529)		Controls (n = 74909)		Rate Ratio	95% CI
Age Groups	No. (Incidence^#^)	Mean follow-up (months)^&^	No. (Incidence^#^)	Mean follow-up (months)^&^		
> = 18, <30	3 (0.58)	43.99	3 (0.19)	62.33	3.05	(0.65–15.10)
> = 30, <40	18 (1.35)	62.01	32 (0.74)	48.06	1.83	(1.03–3.25)
> = 40, <50	64 (1.62)	59.79	115 (0.89)	56.45	1.82	(1.34–2.47)
> = 50, <60	107 (1.80)	56.43	197 (1.02)	57.11	1.77	(1.40–2.24)
> = 60, <70	129 (2.40)	62.51	330 (1.92)	57.29	1.25	(1.02–1.53)
> = 70	58 (2.23)	60.09	198 (2.37)	59.08	0.94	(0.70–1.26)
Total	379 (1.92)	59.79	875 (1.37)	57.22	1.40	(1.24–1.58)
Age-adjusted standardized incidence ratio					1.40	(1.24–1.58)

Those who had a diagnosis of bronchiectasis, TB_attacher, COPD, or asthma were excluded.

#: the incidence was estimated by per 1000-person-year.

&: The mean month of follow-up was estimated from those with onset of TB.

**Table 3:** The age-adjusted standardized incidence ratio of incident tuberculosis among male diabetic patients stratified by age.

	Type 2 diabetes (n = 25516)		Controls (n = 79315)		Rate Ratio	95% CI
Age Groups	No. (Incidence^#^)	Mean follow-up (months)^&^	No. (Incidence^#^)	Mean follow-up (months)^&^		
> = 18, <30	4 (0.79)	32.48	13 (0.80)	41.48	0.98	(0.32–3.01)
> = 30, <40	42 (2.01)	51.11	60 (0.91)	52.69	2.21	(1.49–3.28)
> = 40, <50	154 (2.69)	57.89	225 (1.26)	57.83	2.14	(1.74–2.62)
> = 50, <60	156 (2.65)	63.02	313 (1.70)	59.25	1.56	(1.29–1.89)
> = 60, <70	160 (4.25)	54.35	407 (3.45)	63.59	1.23	(1.03–1.48)
> = 70	127 (7.00)	56.22	347 (5.80)	58.84	1.21	(0.99–1.48)
Total	643 (3.25)	57.32	1365(2.19)	59.75	1.48	(1.35–1.63)
Age-adjusted standardized incidence ratio					1.48	(1.35–1.63)

Those who had a diagnosis of bronchiectasis, TB_attacher, COPD, or asthma were excluded.

#: the incidence is estimated by per 1000-person-year.

&: The mean month of follow-up was estimated from those with onset of TB.

Table 4 shows the hazard ratio of the occurrence of TB among all of the participants after adjusting for potential confounders, such as age, sex, COPD, asthma, bronchiectasis and type 2 diabetes. The factors significantly associated with incident tuberculosis were age (HR = 1.04, 95% CI = 1.04–1.04), male gender (HR = 1.83, 95% CI = 1.73–1.94), bronchiectasis (HR = 2.36, 95% CI = 2.11–2.63), COPD (HR = 1.37, 95% CI = 1.23–1.39) and asthma (HR = 1.12, 95% CI = 1.04–1.20). Type 2 diabetes (HR = 1.31, 95% CI = 1.23–1.39) was significantly associated with incident tuberculosis after adjusting age, sex, bronchiectasis, COPD, and asthma.

**Table 4:** The hazard ratios of confounders for Tuberculosis.

	Tuberculosis		Hazard Ratio (95% CI)		Adjusted Hazard Ratio (95% CI)	
	Yes (n = 5103)	No (n = 248246)				
Age (mean ± SD; years)	61.80±11.67	55.31±12.48	1.04	(1.04–1.04***)	1.04	(1.04–1.04***)
Sex			1.71	(1.62–1.81 ***)	1.83	(1.73–1.94***)
Males	3176 (2.45)	126294 (97.55)				
Females	1927 (1.56)	121952 (98.44)				
Type 2 diabetes			1.32	(1.24–1.40***)	1.31	(1.23–1.39***)
Yes	1553 (2.44)	62177 (97.56)				
No	3550 (1.87)	186069 (98.13)				
COPD			2.34	(2.18–2.51***)	1.37	(1.26–1.48***)
Yes	964 (4.85)	18895 (95.15)				
No	4139 (1.77)	229351 (98.23)				
Asthma			1.49	(1.39–1.59***)	1.12	(1.04–1.20***)
Yes	1198 (3.16)	36667 (96.84)				
No	3905 (1.81)	211579 (98.19)				
Brochiectasis			3.30	(2.96–3.67)	2.36	(2.11–2.63***)
Yes	363 (7.32)	4597 (92.68)				
No	4740 (1.91)	243649 (98.09)				

*** : p<0.001.

The hazard ratios of two comorbidities interactions (type 2 diabetes, COPD, asthma, and bronchiectasis) for incident Tuberculosis are shown in Table 5. Patient with both COPD and asthma were found not to be at significantly higher risk of incident tuberculosis. However, all patients with a combination of other to comorbidities (type 2 diabetes, COPD, asthma, and bronchiectasis) were found to be a significantly greater risk of incident tuberculosis after adjusting for sex and age.

**Table 5:** The hazard ratios of two comorbidities interactions (type 2 diabetes, COPD, asthma and bronchiectasis) for incident Tuberculosis.

	COPD	Asthma	Brochiectasis
Type 2 Diabetes	1.25 (1.11–1.42)^#^	1.13 (1.01–1.26)*	1.83 (1.47–2.26)^#^
COPD		0.96 (0.88–1.06)	1.57 (1.33–1.85)^#^
Asthma			1.54 (1.32–1.79)^#^

The Hazard ratios were estimated after adjustment of sex and age.

* : p<0.05; #: p<0.001.

**Figure 1:**
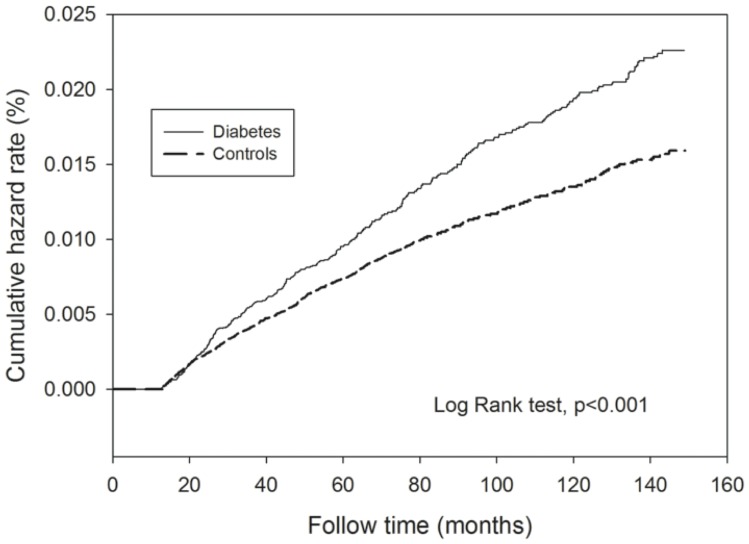
The cumulative hazard rate of incident TB in female diabetic patients and controls.

**Figure 2:**
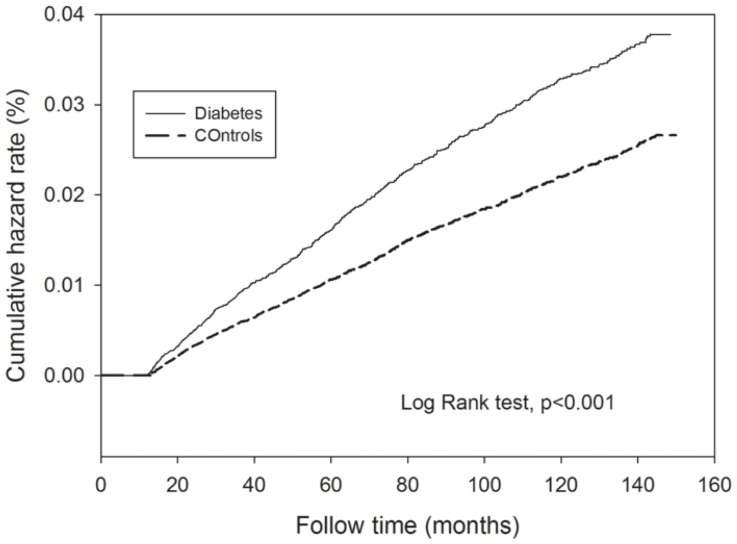
The cumulative hazard rate of incident TB in male diabetic patients and controls.

Figure 1 and Figure 2 depict the cumulate hazard rates for TB for diabetes patients compared to the controls among females and males. Both male and female patients with type 2 diabetes had significantly higher risk of incident tuberculosis compared to the controls (p values were less than 0.001 estimated by Log-Rank test.

## Discussion

In this large, population-based cohort study, patients with Type 2 diabetes (HR:1.31, 1.23–1.39, p<0.001) were found to have a significantly higher risk of developing tuberculosis after adjustments for possible confounding factors such as age, gender, COPD, asthma, and bronchiectasis. Relative risks of developing tuberculosis among patients with type 2 diabetes were higher in younger age groups, but somewhat lower in older age groups.

One meta-analysis [4] reported that odds ratios for the impact of diabetes on tuberculosis ranged from 1.16 to 7.83 depending on study quality variables and differences in population studies. A cohort study in Hong Kong, limited to people aged 65 years or more, found an adjusted hazard ratio (HR) of 1.77 for active tuberculosis. Kim et al. [10] found that diabetic patients were at relative risk of 3.47 for pulmonary tuberculosis in an unadjusted analysis among Korean civil servants. Dobler et al. [11] reported that people with diabetes had a 1.5 fold increased risk of developing tuberculosis in a large population-based cohort study conducted in Australia. The results of our study showing that patients with type 2 diabetes is a moderated risk for tuberculosis (HR:1.31, 1.23–1.39, p<0.001) are consistent with the findings of previous literature [4], [10], [11]. Recently, Lee et al. [12] also found that diabetic Taiwanese were at relative risk of 1.29 for developing tuberculosis after adjustment of confounding factors. We estimated that type 2 diabetes accounted for 3.03% and 4.09% of incident tuberculosis in female and male population in Taiwan. In the sub-population of patients with type 2 diabetes, our calculations indicated that type 2 diabetes accounted for 28.6% and 32.6% of incident tuberculosis in female and male with type 2 diabetes. Of note, ours is the only prospective study on type 2 diabetes and incident tuberculosis conducted in a general population and controlled for sex, age, and potential major confounders.

Tuberculosis continues to be a major global health problem. Current TB control measures focus on the prompt detection and treatment of those with infectious forms of the disease to prevent further transmission of the organisms. Despite the enormous success of this strategy in TB control, the incidence of TB is declining slowly globally, at less than 2.2% annually [13]. Additional preventive steps including addressing risk factors that increase the individual’s susceptibility for incident TB, multi-drug resistant TB and relapse after treatment should be taken. Diabetic patients with tuberculosis often present with lower lung infiltrates and more cavitary lesions [5]. One systemic review [14] reported an association between diabetes and an increased risk of failure and death during tuberculosis treatment. Higher rates of multidrug resistant tuberculosis infection have also been found in diabetic patients as compared to non-diabetic subjects [15], [16]. Diabetes associated with tuberculosis increased the risk of relapse after successful completion of anti-TB treatment [11]. Previous studies [17], [18] found that patients with diabetes and tuberculosis had a higher bacillary load in sputum, which might increase the risk of spreading of tuberculosis.

Our study found that patients with type 2 diabetes were at high risk for incident tuberculosis especially in younger ages. As the number of people with type 2 diabetes increases globally, the age of onset of type 2 diabetes is decreasing. In light of our findings, type 2 diabetes may pose an even greater challenge to tuberculosis care and control in the years to come. In spite of these findings, the potential public health risk and clinical importance of this relationship seems to be largely ignored. National clinical and policy guidelines in the UK on the control of tuberculosis does not consider its relationship with diabetes [19]. The WHO’s new “Stop TB strategy” refers to the problem of TB in “high risk groups” including people with diabetes [20], but WHO has not yet made specific recommendations concerning the relationship between these two conditions. The recently published international standards for TB care give only cursory mention of diabetes [21], [22]. There is an urgent need for new research to guide policy and practice in this area to promote the decrease in tuberculosis.

The mechanisms by which type 2 diabetes increases susceptibility to tuberculosis are not yet well understood. Leung et al. [23] found that patients with poor recent glycemic control (HbA1c of more than seven percent) were at significantly increased risk of tuberculosis, while those with HbA1c <7% were not. Alisjahbana et al. [24] found an association between impaired fasting glucose patients and tuberculosis (OR: 4.2, 95% CI: 1.5–11.7) similar to that found in patients with type 2 diabetes (OR: 4.7, 95% CI: 2.7–8.1). These findings suggest that hyperglycemia may increase the risk of developing tuberculosis. Some studies [25], [26] have found that T-helper 1 type cytokines are upregulated in M. tuberculosis-infected mice with streptozotocin-induced diabetes and in diabetic patients with increasing levels of glycemia. However, Vallerskorg et al. [27] reported that in diabetic mice there is an initial delay in the adaptive immune response, including the appearance of IFN-r-producing T cell, the dissemination of bacteria from lung to lymph nodes, and the aggregation of lymphocytes at the site of infection. Such an impaired and altered immune response may also increase susceptibility to infection with multi-drug resistant strains. Non-enzymatic glycosylation of tissue proteins or diabetic autonomic neuropathy may induce changes in cough threshold and alterations in bronchocillary functions; abnormal basal airway tone and reduced bronchial reactivity may also contribute by increasing stagnation and reducing clearance of secretions and bacterial load [28]. The above findings suggest that the strong association between type 2 diabetes and tuberculosis may primarily emerge as a result of hyperglycemia and its consequences on cellular functions involving physical barriers and immune defenses.

Our study found age to be a strong risk factor for tuberculosis. We also found a stronger association of type 2 diabetes and tuberculosis in people under the age of 40 years old and declining rate ratio in those over 40 years old. The studies by Kim et al. [29] and Ponce-de-Leon et al. [30] also found that estimated varied markedly by age, with substantially higher estimates among younger people. These two studies did not distinguish between type 1 and type 2 diabetes, so it is possible that younger people with diabetes might have had type 1 diabetes, a more severe form of diabetes with a juvenile onset. Another study, based on diabetes diagnosis based on medical records, did not show the same trend for age [31]. However, our study excluded patients with type 1 diabetes and our diagnosis of type 2 diabetes was based on clinical diagnosis and follow-up prescriptions of anti-diabetes medications. Our results revealed that elderly controls may have had an elevated risk of tuberculosis compared to younger ones especially those more than 70 years old, thus reducing the apparent effect of type 2 diabetes. In our study, the incidence of tuberculosis was even higher in diabetic men (2.01 vs. 1.70 per 1000-person year) and women (1.35 vs. 1.02 per 1000-person year) in the third decade as compared to non-diabetic subjects in the fifth decade of life. As the number of young onset type 2 diabetes increases globally, type 2 diabetes might adversely affect tuberculosis care and control. Therefore, preventive measures including those that address the possibility that type 2 diabetes that increase the individual’s susceptibility for incident TB should be taken to further reduce the incidence of tuberculosis.

Our study found type 2 diabetes, COPD, asthma, and bronchiectasis to be significantly associated with incident tuberculosis by cox regression analysis. The interactions of these predictors were analyzed. Except for patients with both COPD and asthma, patients with two of these comorbidities were found to be a significant risk of incident tuberculosis (Table 5), though there were no significant interactions between type 2 diabetes, COPD, asthma, and bronchiectasis. It is unclear why there was no significantly increased risk of incident tuberculousis in patients with both COPD and asthma. Further research is needed to explore this.

This study has some limitations. There was no individual information, such as body mass index, tobacco use, lifestyle, or no laboratory results available in the claims database. Therefore, we could not extrapolate the association between incident tuberculosis and other metabolic profiles as found by other investigators, though we tried to collect the associated risk factors of tuberculosis including COPD, asthma, and bronchiectasis.

In conclusion, type 2 diabetes was found to be an independent risk factor for incident tuberculosis after adjusting for potential confounders in this prospective cohort study in general population. The current diabetes epidemic may lead to a resurgence of tuberculosis in endemic regions. Continuing to ignore or underplay this association may undermine and undo decades of painstaking gains in TB control.
